# Messaging strategies for communicating health-related information in social media—a content and effectiveness analysis of organ donation posts on Instagram in Germany

**DOI:** 10.1186/s12889-023-15736-2

**Published:** 2023-05-11

**Authors:** Alexandra Olsacher, Celina Bade, Jan Ehlers, Bettina Freitag, Leonard Fehring

**Affiliations:** 1 Witten/Herdecke University, Faculty of Health, School of Medicine, Alfred-Herrhausen-Straße 45, Witten, 58455 Germany; 2 Witten/Herdecke University, Faculty of Health, School of Medicine, Didactics and Educational Research in Health Care, Witten, Germany; 3 Helios Universitätsklinik Wuppertal, Klinik Für Gastroenterologie, Hepatologie, Endokrinologie Und Diabetologie, Wuppertal, Germany

**Keywords:** Organ donations, Health communication, Social media, Message effectiveness, User engagement, Content analysis

## Abstract

**Background:**

Although organ transplantation is a very effective clinical solution to save the lives of patients suffering from organ failure, the supply of donated organs still cannot meet its growing demand. Educating the society about organ donation is a critical success factor in increasing donation rates, especially in countries that require potential donors to proactively register and opt-in (e.g., Germany). While social media has emerged as an effective tool for disseminating health information, recent evidence suggests that published organ donation content (both online and offline), aimed at raising awareness, still lacks effectiveness. To develop recommendations for optimizing organ donation messaging via social media, this study not only examines the current state of organ donation communication on Instagram, but also identifies factors that contribute to message effectiveness.

**Methods:**

We conducted a retrospective content analysis to in-depth assess organ donation-related content published on Instagram in Germany between January and March 2022. Systematic coding allowed to identify common themes, sentiments, and communication strategies, which were analyzed for their effectiveness using linear regression analyses.

**Results:**

Of the 500 organ donation posts, 57% were published by institutional authors while the remainder was shared by private accounts. Most content was aimed at the general population and shared neutral (80%) or positive sentiments (17%). Transformative messages, positive emotions, posts published by the transplant recipient and the image of a human served as predictors for post effectiveness measured in terms of likes (*p* < 0.001) and comments (*p* < 0.01). Sharing personal experiences (*p* < 0.01) and highlighting the meaning of organ donations (*p* < 0.05) resulted in significantly higher audience engagement than any other topic discussed.

**Conclusion:**

Our findings highlight the need for health officials to work closely with organ transplant recipients to publicly advocate for organ donations by sharing personal and transformative messages. The high share of posts published by transplant recipients indicates a certain openness to share personal experiences with broad audiences. Different message characteristics served as predictors for message effectiveness (i.e., increased audience engagement) which can likely be extrapolated to other health-related use cases (e.g., cancer screening).

**Supplementary Information:**

The online version contains supplementary material available at 10.1186/s12889-023-15736-2.

## Introduction

### Background

Organ transplantations are a highly effective clinical solution [1] to save lives of patients with terminal organ failures [2, 3]. In 2021, about 28,000 organs were transplanted in the European Union (EU), with kidneys, livers, hearts and lungs being the most common [1, 4]. Despite new technological innovations in the medical field that increase the success rates of organ transplantations and the number of patients benefitting from them [5], the supply of donated organs still cannot meet the demand [1]. In 2020, 21 people in the EU have died every day waiting for an organ transplant while a new patient was added to the waitlist every ten minutes [4].

**Fig. 1:**
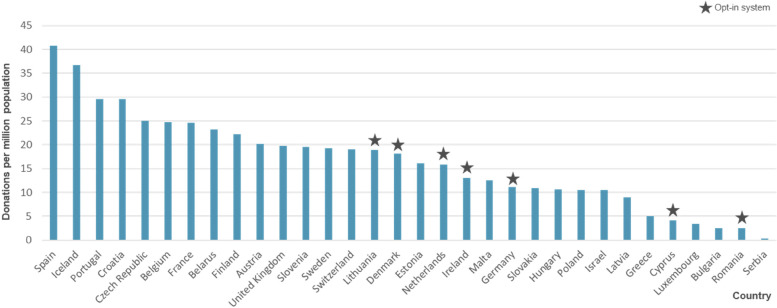
Organ donations (excluding living donations) per million population in 2021 [12, 13]

Transnational institutions such as the European Kidney Alliance (EKHA) aim to collectively increase organ donation rates in the EU, but success rates vary significantly within countries. [6]. Moreover, countries can be differentiated according to the organ donation system (i.e., opt-in versus opt-out) applied [6]. By default, opt-out systems consider everyone to be a potential organ donor unless the person has expressed explicit opposition before death [7]. Opt-in systems require explicit consent from the patient or their relatives [8, 9]. Prior research on the effect of the organ donation system on donation rates has found mixed evidence [6], some stating a positive effect of an opt-out legislation [8, 10] while others find no effect of the organ donation system on donor rates [6, 7]. Germany, a country with an opt-in system, lags behind other EU members in terms of organs donated per capita as shown in Fig. 1 [11].

Educating the public about the importance of organ donations is a critical success factor in increasing donation rates [14–16], particularly in opt-in countries where the public has to make an active decision on their donor status [17]. To inform the population about the importance of organ donations, Germany recently passed a new law asking physicians to inform patients about the possibility of becoming an organ donor [18]. Hereby, the legislation aims to address existing structural deficiencies [18] but may not reach its full potential to address all parts of the society equally, as most patients who seek medical attention are elderly people [19, 20].

### The power of social media for health communication

Numerous studies highlight the importance of social media for sharing health information [21], not only regarding epidemic control and surveillance [22] but also to raise awareness about blood [23] and organ donations [24]. Currently, 50% of social media users already adjust the way they deal with their health based on information received via online platforms [22]. Especially, Millennials and Generation Z show a high level of trust in social media content [25, 26], which will make it an even more important source of information in the future. In 2017, 25% of surveyed young adults already believed that social media can provide them with useful health information, and half of them actively shared information on their health with the online community [27]. Not only trust but also time spent on social media is expected to increase over the few next years, beyond the roughly 2.5 h currently spent on an average day on any available platform [28].

### Effective social media messaging strategies

As social media is an effective tool to distribute information to large audiences by speeding up and enriching communication [29], vast research has investigated factors contributing to social media message effectiveness [30, 31]. Higher levels of information [32], creativity [33] and emotion provided in advertisements increased user engagement across social media platforms (e.g., giving likes or sharing content) [34]. The theory of ‘transformational’ and ‘informational’ advertising suggests that human beings purchase products for either emotional (hedonic) or rational (utilitarian) reasons [35]. Hedonic or emotional appeals – also referred to as transformational advertising—rely on creating experiences for the potential customers (e.g., make them feel happy about a product) [35, 36] that influence people’s behavior [33] and make the content memorable [37]. Utilitarian or rational appeals – also referred to as informational advertising—are messages that inform people about the functional or rational benefits of a product [35, 36, 38]. Not only the messaging strategy (i.e., emotional versus rational appeals) determine whether a message is effective, but also the type of product displayed. Based on existing marketing research, informational advertisements are preferred for utilitarian products (i.e., functional and necessary products) and transformational advertisements for hedonic products (i.e., enjoyable, luxurious products) [39, 40]. Building upon this theory, one can consider organ donations, more specifically the donated organ, to be an utilitarian good (i.e., a lifesaving necessity) from the perspective of the transplant recipient and “society as a whole”. Moreover, from an utilitarian perspective, posthumous organ donations might be seen as the rationally, best action as they contribute to the best outcomes for everyone (i.e., lives being saved) [42]. Nonetheless, similar to blood donations, that are motivated by utilitarian as well as altruistic reasons [41], organ donations may also be motivated by altruism. From the perspective of a deceased organ donor, who does not directly benefit from the organ donation, it might be considered a hedonic good. Based on these assumptions, we aim to test whether organ donations will be more susceptible to informational or transformational advertising.

### Current studies on organ donation message effectiveness on social media and research gap

Efforts to raise awareness about organ donation via social media platforms have—in experimental research—been proven successful in increasing target audiences’ willingness to donate organs [43] and the likelihood of registering as a donor [44]. A few strategies are particularly effective such as i) creating relevancy among the target audience (e.g., illustrating how message recipients or their close network may one day benefit of donations) [44], ii) revealing the recipient’s identity (e.g., illustrating a picture or name of the patient receiving the organs) [43] and iii) leveraging celebrity testimonials [45].

Despite theoretical knowledge about effective organ donation messages [43, 44, 46], past studies in different cultural settings identified a gap between messages displayed on (social) media and the most effective messages to increase organ donations [47, 48]. An assessment of 14 offline, public organ donation campaigns in Germany concludes that organ donation posters have little effectiveness and, in particular, cannot motivate undecided or donation-skeptical people to become active donors [49]. Celebrity endorsements which are known to be effective in providing health [45] and organ donation information [50] are underutilized: Celebrity advertisements, for example in the United States, account for only 10% of organ donation messages across media channels [51]. Likewise, previous work on organ donation messages on Chinese micro-blogging platform (i.e., Weibo) finds a mismatch between effective organ donation themes (i.e., ‘*statistical descriptions of organ donations’* and ‘*meaningfulness of donations’)* and most frequently communicated themes (i.e., ‘*organ donation behaviors’)*. Although the study provides important insights into organ donation information, it is unclear whether these findings are transferrable to other cultures and channels [48]. Negative media coverage, on the other hand, can also have significant effects on organ donation rates for example when organ donation scandals become public. Röck et al., (2017) found that the number of organ donors dropped significantly after a transplantation scandal was uncovered in Germany which not only highlights the need to adhere to professional and ethical standards [52] but also the importance of effective media campaigns to make up for negative press.

Most recent research on organ donations and social media has focused on micro-blogging platforms such as Twitter [53, 54] or Weibo [48], probably because data is more readily available (i.e., API downloads) for quantitative research [55]. However, studies assessing public health information on picture-sharing platforms (e.g., Instagram, Pinterest) are still scarce. Recent research calls for more insights on how best to use social media as a tool for health interventions [55] and what creative means (e.g., visualizations, types of narratives) should be included in health communication [56].

To our knowledge, no comparable study has yet qualitatively and quantitatively assessed the availability and effectiveness of organ donation information shared on the picture-sharing social media platform Instagram in Germany. In this research, we focus on two research questions: First, we aim to understand which organ donation information and campaigns are available on Instagram through a systematic content review. Second, we will examine the effectiveness of different messaging strategies, characteristics, and organ donation themes to derive practical implications for communicating organ donation, and possibly also other health-related topics aimed at raising public awareness.

## Method

We conducted a retrospective content analysis of 500 Instagram posts to understand the prevalence of organ donation posts and their effectiveness. Our study design most closely resembled Selzer et. al.’s research (2017), who analyzed 500 publicly available Instagram posts using criterion sampling [57].

### Data collection

All Instagram posts with the hashtag #organspende (translated to English: #organ donation) that were posted from January 1 to March 7, 2022 were extracted using a criterion sampling approach [58]. We collected a sample of 500 posts, which exceeded the sample size of similar retrospective content analyses on Instagram [48, 59, 60]. We selected only posts in German language and excluded all duplicates and posts related to Swiss or Austrian content to limit the search to a geographical area.

As no legal API download was available at that time (caused by Instagram’s restrictive data regulations), we collected all Instagram posts manually. Due to the dynamic nature of social media, we collected the data via screenshots, similar to e.g., Gabarron et. al. [61] and Carotte et. al. [62], and saved it on a local drive. These screenshots allowed a static sample to be further analyzed. Additional information such as the source of the posts (i.e., author), number of likes and comments were manually recorded using Microsoft Excel.

### Establishing the codebook

We developed a code book in Microsoft excel to record the categories of interest for each post. A first draft was built based on results of previous research, collecting binary variables (e.g., transformational versus informational [35], picture versus text [61], message sentiment [57] etc.) and key organ donation themes previously discovered on social media. For the latter, we classified the content topics into 5 categories: (1) personal experience, (2) meaning of the donation, (3) statistical description, (4) issues and policies with donations, and (5) organ donation knowledge [48]. The codebook was tested by the authors with 50% (*n* = 250) of the organ donation posts. In case a post could not be allocated to one existing topic, a new category was added to ensure the manual was suitable for the type of social media channel (i.e., Instagram) and country of interest selected (i.e., Germany). Three new categories emerged during this iterative process: (6) donation awareness, (7) merchandise/run, and (8) other. Detailed instructions for the coding exercise (incl. examples of coded themes) are provided in supplementary material 1.

### Data coding

Two independent coders were trained on a subsample of 50 organ donation posts to ensure interrater reliability, measured using Cohen’s kappa, which is commonly used if two independent coders are present. For any coded variable (i.e., transformational/informational, message sentiment and key organ donation theme) that showed inter-coder reliability below 0.6 (> 0.60 indicating at least ‘substantial agreement’ according to Cohen’s kappa classification [63]), we resolved any controversy using the Delphi method [64]. Using this method, consensus was reached by engaging the author and the independent coders in a discussion.

### Data analysis

We performed descriptive statistics in Microsoft excel to identify the posts’ authors and most common organ donation themes discussed. To measure the posts effectiveness, we used the audience’s engagement as a proxy which is well recognized in social media marketing research [33, 65]. We calculated the likes-to-follower ratio (Eff_post_l = number of likes_post/number of followers_account) as well as the comments-to-follower ratio (Eff_posts_c = number of comments_post/numbers of followers_account) to control for different numbers of followers per account (i.e., people being exposed to the post). As leveraged in previous academic research, this is an effective way to make post effectiveness (i.e., number of likes) comparable across multiple Instagram accounts, regardless of follower count [32, 55, 65]. We used linear regression models to analyze, the relationships between the independent variables (IV) (i.e., transformational/informational, image of a human, author of post, sentiment, content themes) and dependent variables (DV) (i.e., likes-to-follower ratio and comments-to-follower ratio). The effect size for each variable was determined using Cohen’s d [66]. For the linear regression analysis, we used the statistical software ‘Stata version 17’ with the significance level defined as *p* < 0.05.

## Results

Within the studied period (January 1 to March 7, 2022), a total of 593 Instagram posts with the hashtag #organspende (translated to English: #organ donation) were identified. 93 posts, which included duplicates or showed irrelevant content were removed. On average eight posts were published per day during the sample collection period after excluding duplicates and not relevant postings.

### Descriptive statistics—Authors of organ donation messages

As shown in Table 1, most posts were published by institutional authors (57%), such as governmental organizations, hospitals, social associations, news stations or small businesses and aimed at the general population (97%) rather than health care professionals. Meanwhile celebrities posting about organ donations or sharing experiences played a minor role (< 1% of posts). The remaining proportion of posts (43%) was published by individual authors (e.g., mother telling the audience that her son has received a new organ transplant).

**Table 1:**
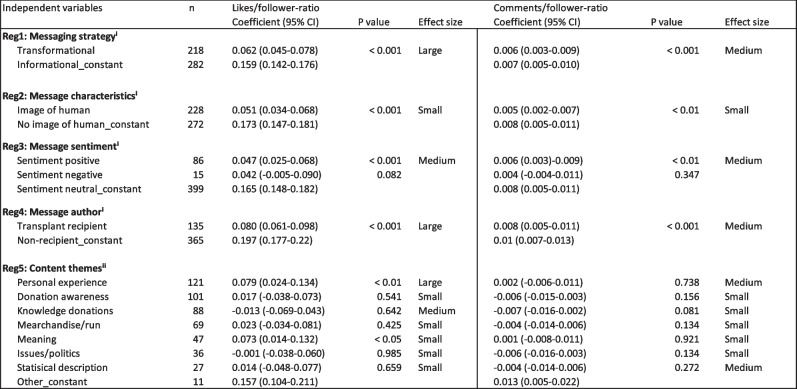
Descriptive statistics of results

### Critical role of transplant recipients

Organ recipients aimed to raise awareness about organ donations by sharing their personal experiences via Instagram. Almost one third of posts (27%) were published by the organ recipient or, in the case of small children being affected, by the recipient’s parents. Only 1% of organ donors (i.e., living donors) or families of organ donors actively promoted the topic via Instagram. All remaining posts (72%) were shared by a third party who was neither the donor nor the recipient of an organ transplant.

### Sentiments range from predominantly neutral to positive

Instagram posts on organ donation mostly shared neutral (80%) or positive sentiments (17%) such as gratitude for a new transplant or joy about successfully managing a disease. Only 3% of posts shared a negative sentiment such as anger about donation laws and politics or sadness about diseases that require organ transplantations.

### Personal experiences and donation awareness as major themes

Among the eight identified organ donation themes most discussed ‘personal experiences of donations’ (25%) and ‘donation awareness’ (20%) (i.e., authors sharing that they signed up for a donor card). The theme ‘knowledge on donations’ (18%) was the third most frequent topic discussed.

### Regression analyses – message strategy, characteristics, content published by transplant recipients and themes as predictors for higher effectiveness

Transformational messages, positive sentiment, posts shared by transplant recipients, and image of a human increased the effectiveness of organ donation posts in terms of likes (*p* < 0.001) and comments (*p* < 0.01) in the linear regression analyses. As shown in Table 2, in each of the linear regressions, results remained significant (*p* < 0.01) when controlling for gender of the author as well as the size of the follower base. We found a large effect size (0.44) for the posts’ effectiveness (i.e., likes ratio) and type of message strategy (i.e., transformational) when applying Cohen’s d [66].

**Table 2:**
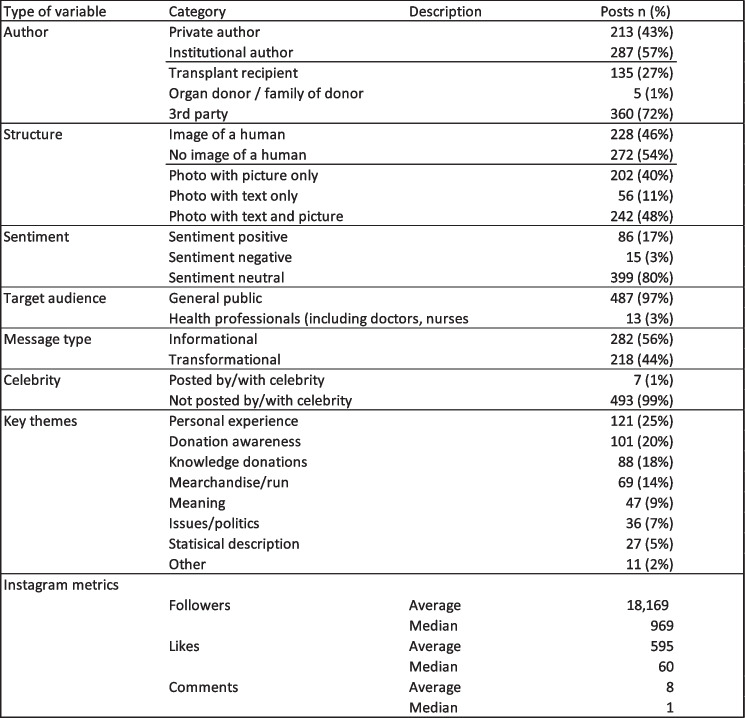
Regression analyses

### Increased engagement (i.e., likes) through messages highlighting personal experiences and meaning

Two of the eight reported organ donation themes, ‘personal experiences’ (*p* < 0.01, effect size large (0.59)) and ‘meaning for the organ donor’ (*p* < 0.05, effect size small (0.18)), showed a positive association with the posts’ effectiveness measured as likes-to-follower ratio in the multiple linear regression model. Both themes were closely related as they showed either the purpose of an organ donation for society, recipients, or donors or personal experiences with organ transplantations.

## Discussion

This study examined not only how social media picture-sharing platforms are currently leveraged to spread the knowledge about organ donations in an opt-in country with low organ donation rates (i.e., Germany), but also how post effectiveness (using user engagement as a proxy [33, 65]) varies depending on the message type. Our results confirm that different messaging characteristics (i.e., transformational, positive sentiment, image of a human) and content themes serve as predictors of higher audience engagement. We have, thus, built on Vanholder et al.’s call for further research on comparing educational organ donation campaigns (aimed at the general population) to derive practical implications [6].

### The crucial role of transplant recipients as advocates for organ donation awareness

Our results support the practicality and importance of inviting organ transplant recipients to advocate for organ donations by sharing personal experiences. Some organ transplant recipients seem open to share their stories with a wider audience (i.e., the Instagram community), as almost one third of the posts was published directly by organ transplant recipients. We, therefore, shed some light on the question raised by Harel et. al. (2017) on whether organ transplant recipients are willing to share experiences with a wider audience [43]. What could potentially contribute to the willingness to share personal information are the specific characteristics of the platform (i.e., social media) studied. Since social media has caused a fundamental shift in the way people communicate today [67], people nowadays might be more open and trusting to share personal information [25] which could be leveraged when designing health campaigns on social media. While our study identified the relative share of posts revealing the organ recipient’s information out of the collected sample, it does not provide an answer about the willingness to share personal information of all patients who have ever received a transplant.

Sharing personal experiences and highlighting the meaning of organ donations received significantly higher engagement scores (i.e., likes ratios) than any other topic discussed which is consistent with previous qualitative and experimental studies. For example, disclosing the identity of the organ transplant recipient increased willingness to register as an organ donor [47], since reading about a person who has received a transplant induces thoughts of saving lives, as opposed to reading information about a deceased donor [43]. These findings seem to apply to other types of social media (i.e., micro-blogging) and cultural settings (i.e., China). Previous research on organ donations conducted on the social media platform Weibo in China confirms that creating meaning for the audience is most influential to drive community engagement [48]. Interestingly, the term ‘meaning’ was interpreted differently in the social media posts in the Chinese sample, where posts emphasized the meaning of organ donations from the perspective of the donor (or the donor’s family) [48]. In Germany, on the contrary, the ‘meaning’ was almost exclusively emphasized from the recipient’s point of view which might be due to the impact of different cultures on attitudes and behaviors [68] (i.e., sharing content on social media). For example, individualistic cultures (i.e., Germany [69]) value self-enhancement, which may be reflected in highlighting the importance of a donation to the recipient [68]. Cultures with low levels of individualism (i.e., collective cultures such as China [69]) in contrast value universalism which is defined as caring for the welfare of others [70]. This value may be reflected in posts that highlight the importance of donations to the organ donor (or donor’s family) as they demonstrate that they have contributed to the well-being of others. Nonetheless, further work is needed to unravel various aspects of culture on social media organ donation posts. Moreover, this paragraph already briefly touches upon the question on how different messages and content themes are perceived by the audience in a given cultural context. Another topic of high relevance is nonetheless the target audience’s actual motivation to donate (e.g., being altruistic, in need of a donor organ, trusting the system or wanting to save someone’s life) [71] or believes which likely influence the way they perceive the organ donation message. While cultural differences and motivators for donation may explain parts of the differing attitudes, another factor to consider is the individual’s religion, which might be for (e.g., Christianity or Hinduism) or against (e.g., ultraorthodox Haredim) organ donations [72]. Against this backdrop, future research will benefit from a more nuanced approach of for example examining organ donation message effectiveness in relation to the audience’s underlying attitudes, norms and believes as previously suggested by Noar et. al. (2007) in the context of public health messaging [73].

### Messaging strategies and characteristics as predictors of higher post effectiveness

We identified three messaging features which predict higher chances of receiving likes or comments. First, organ donation messages with transformational content, that is known to trigger emotions in audiences [33, 37], were more effective in increasing engagement than posts providing informational content. Our results are consistent with a previous study that analyzed Facebook posts from Fortune 500 companies for their messaging strategy and effectiveness [35] and a study that evaluated Facebook posts from well-known corporate brands [74]. As Tafesse et al. (2018) argue, transformational posts may have a higher likelihood to foster consumer transformation because they leverage emotional and hedonic signals [74] that are known to positively influence content transmission [75]. Second, messages with a positive sentiment motivated a higher number of people to react to a post. This is not surprising, as social media posts that induced positive emotions (e.g., make someone ‘look good’ or make someone ‘feel happy’) were found to be more engaging to the audience and led to a higher likelihood of being shared with others [75, 76]. Third, the visual illustration of a human in the post had a positive impact on likes and comments ratios. Our results are consistent with previous scientific research, which states that images in the vicinity of text increases the readers’ attention to the information provided [77] (e.g., when receiving instructions on wound care [78]). Additionally, images of humans have enhanced advertisement effectiveness [79, 80] and overall engagement with social media content in a health context (i.e., anti-vaping campaigns) [81]. Previous research on public health media campaigns has shown that the effectiveness of such campaigns varies not only with the messaging strategy chosen, but also with the topic covered [48, 82]. In summary, our research thus contributes to existing literature as well as the imperative that health campaign messages should be designed based on scientific research [82] by identifying how certain messaging characteristics impact the effectiveness of organ donation campaigns. As we found some similarities to effective campaigns in other contexts (e.g., commercial or public health), we argue that public health authorities could learn from these existing campaigns that also employ the characteristics identified in this research.

## Limitations

Our research has some limitations which result from the methods applied. First, since no API download was available to collect and analyze posts automatically, we were limited to a rather small sample that could be analyzed manually. Future research could try to find solutions to increase sample size. Second, we used audience engagement (i.e., number of likes or shares) as a proxy for post effectiveness as done in similar studies [33, 65]. Previous research has found a strong link between social media interactions and message effectiveness [83, 84]. Nevertheless, our research cannot establish a causal link between organ donation message characteristics and actual organ donation behavior. Further studies could explore this relationship in experimental settings. Third, we used the follower base of the Instagram account which published the organ donation post to make the posts’ effectiveness comparable to each other. Adjusting for the number of interactions (i.e., likes) by the number of followers has been leveraged in previous studies [32, 55] but still has potential limitations. Instagram post can be not only read by the account followers, but also by people following a specific hashtag (e.g., ‘organ donations’). Therefore, additional people (i.e., non-followers) could have been exposed to a post, reducing post effectiveness. Fourth, given the method applied (i.e., manual collection of posts), we were not able to control for the composition of the account’s follower base (e.g., followers being friends or family with the author who might be more sensitized for organ donations). Therefore, one has to acknowledge that there may be a correlation between the follower composition and the absolute effectiveness of the post, which becomes particularly relevant when comparing the study results with other social media campaigns (e.g., the number of likes) outside of this study. To mitigate this bias, we do not look at the absolute but rather the relative effectiveness of the posts containing different message characteristics (e.g., comparing posts with versus without the picture of a human). Moreover, all of the discussed findings are robust when looking at small (e.g., private accounts with fewer followers) as well as large accounts (e.g., an NGO’s account with a large follower base). Furthermore, the reach of the authors within our sample, which ultimately attributes to the effectiveness, may significantly differ as private authors have significantly less followers than institutional authors have.

## Conclusion

**Fig. 2:**
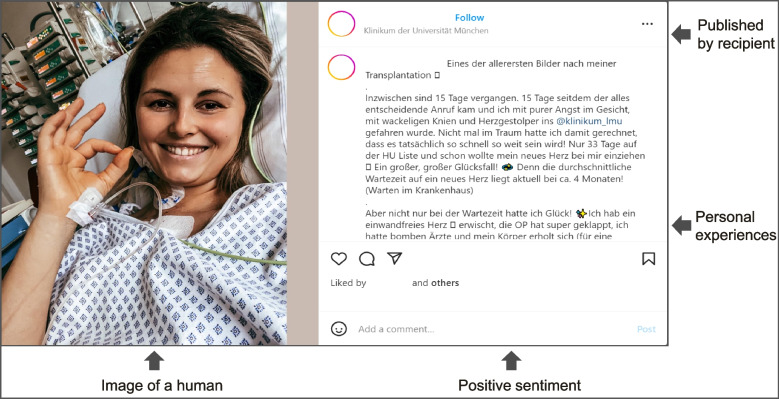
Exemplary Instagram post highlighting predictors of high message effectiveness (permission for re-print received from rights holder) [85]

**Fig. 3:**
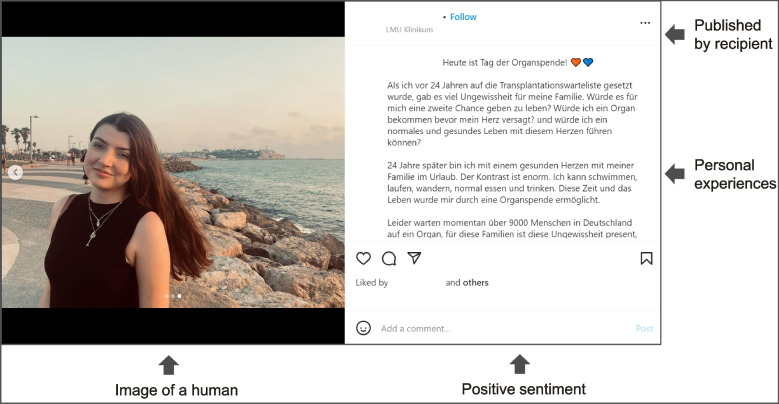
Exemplary Instagram post highlighting predictors of high message effectiveness (permission for re-print received from rights holder) [86]

Considering its increasing importance, social media will lead to a fundamenta shift in health communication by changing the speed and type of interactions between health professionals and patients [67]. We, therefore, believe it is imperative that health authorities make extensive use of social media platforms when designing organ donation awareness campaigns; especially if they are aimed towards the younger generation. In doing so, the effectiveness of the message can be increased by applying the measures (i.e., image of a human, positive sentiment, published by recipient, personal experiences) highlighted in Figs. 2 and 3 as well as intensifying collaboration with transplant recipients.

Although our sample was limited to Germany, we assume that our results can be transferred to other nationalities with comparable value and believe systems which nonetheless requires further research. Moreover, we believe that our study has important implications for other (public) health-related interventions aimed to increase public awareness e.g., cancer screening which is a prerequisite to increase the likelihood of cancer survival (e.g., for ovarian or breast cancer), might be one example [87–89]. Previous research emphasizing the power of social media to disseminate cancer prevention, screening, and treatment messages to large audiences [90, 91], can be complemented by our results to increase message effectiveness (e.g., by asking patients to share experiences and importance of early detection). Nonetheless, our results may be most relevant for digital heath interventions targeting Millennials and Generation Z such as educative vaccination campaigns or information on sexually transmitted diseases. Further research may elaborate circumstances under which our identified messaging strategies support message effectiveness in the context of other health use cases.

## Supplementary Information

**Additional file 1.**

## Data Availability

The raw data collected used for the content analysis of this study is not publicly available due to the sensitivity of the topic (i.e., organ donations) and potential privacy concerns (potential de-anonymization of Instagram posts). However, data can be made available upon request from the corresponding author. The coding manual can be found in supplement 1.

## Abbreviations

EU: European Union
EKHA: European Kidney Alliance
IV: Independent variable
DV: Dependent variable
