# Supplementary material for: Messaging strategies for communicating health-related information in social media—a content and effectiveness analysis of organ donation posts on Instagram in Germany
Source: BMC Public Health. 2023 May 11;23:867. doi: 10.1186/s12889-023-15736-2 (PMC10176822; doi:10.1186/s12889-023-15736-2)
Supplement: Supplementary file 1 — Additional file 1. [file 12889_2023_15736_MOESM1_ESM.docx]

The tables below illustrate which variables have been collected and how each variable is defined. Table 1 summarizes the collected post characteristics while table 2 indicates how each post was coded in terms of sentiment, messaging strategy, and themes.

| **Table 1.** Data collected | Definition & Example (if applicable) | Measure |
| --- | --- | --- |
| Post characteristics |  |  |
| Number of followers | The number of followers indicates the number of people that follow the author of the post. | Count |
| Number of likes | The number of likes shows the interaction of the audience with the post. | Count |
| Number of comments | The number of comments indicates how many people have commented on the content. | Count |
| Date of post | The date on which the post was published on Instagram was recorded in this category. | Date |
| Institutional author | The category ‘institutional author’ shows if the post has been published by an institution (including e.g., government, hospital, NGO, small business). | (yes=1, No=0) |
| Private author | The category ‘private author’ shows if the post has been published by a private person (e.g., mother of a child who has received a transplant). | (Yes=1, No=0) |
| Recipient versus donor or third party | The category records if the post was shared by a transplant recipient, indicated with ‘1’ or an organ donor (including the donor’s family), indicated with ‘-1’. If the post was either shared by a third party or could not clearly be attributed to the recipient or donor category, this was marked ‘0’. | (Recipient=1, Donor =-1, Third Party=0) |
| Image of human | The category ‘image of human’ indicates if a picture of a person was present in the post. | (Yes=1, No=0) |
| Gender of author | The variable ‘gender’ indicates the sex of the author who published the post. If the sex was assumed to be female, this was marked ‘1’. If the sex was assumed to be male, this was marked ‘-1’. The authors collected this variable to the best of their understanding and assigned ‘0’ if the sex was not clearly identifiable. | (Female = 1 Male = -1 Neutral/na = 0) |
| Celebrity | This category records if the post was either posted by a celebrity or included a celebrity in the post. If the answer was yes, this was marked as ‘1’. A celebrity was defined as a famous person known to the majority of the population (e.g., a sports man, a movie star etc.). | (Yes=1, No=0) |
| Target audience: public or professional | This category reflects whether the post addressed the public, which was marked ‘1’ or health professionals (e.g., doctors, nurses), which was marked ‘0’. | (Public=1, Professionals=0) |
| Text description | A short description of the post’s text was captured. | Qualitative description |
| Image description | A short description of the image shared in the post was captured. | Qualitative description |

*Supplementary table 1: Coding of post characteristics*

| **Table 2.** Coding | Definition & Example (if applicable) | Measure | Source |
| --- | --- | --- | --- |
| Categories |  |  |  |
| Sentiment | If the post showed positive emotions (e.g., gratitude, happiness, hope) through text and image, this was marked ‘1’.  For example: A woman smiling into the camera and sharing that she is thankful for receiving an organ transplant.  If the post showed either a neutral tone, mixed positive and negative emotions, or ambiguity, this was marked “0”.  For example: A man shares that he signed up for an organ donor card.  If the post showed negative emotions (e.g., anger, sadness, despair), this was marked as ‘-1’.  For example: A social association highlights how disappointed they are with the low number of registered organ donors in Germany. | (Positive=1, Neutral=0, Negative=-1) | Bouke et al., 2020 [1] |
| Message strategy (transformational versus informational) | If the post attaches an experience to the topic of organ donations and triggered emotions in the coder (either positive or negative), then this was marked ‘1’.  For example: A mother shares how she went to the doctor for a check-up of her son’s heart transplant, which made her feel anxious.  If the post provided factual, rational information to the audience, this was marked as ‘0’.  For example: A news channel posts statistics on the development of organ donor rates in Europe. | (Trans-formational=1, Informational=0) | Cadet, F. T et. al., 2016 [2] |
| Content themes |  |  |  |
| Personal experience | If the post shared personal experiences with organ donations, then this code was marked ‘1’. The category has been slightly adapted from Jiang, X. et al.’s original research and was renamed from ‘organ donation behaviors’ to ‘personal experience’ to more accurately reflect the content themes discussed in our sample.  For example: A girl remembers how her father has received an organ transplant when she was 5 years old. | (Yes=1, No=0) | Jiang, X. et. al., 2019 [3] |
| Donation awareness | If the post either shares information about organ donor cards (e.g., how to register for donations), or explicitly asks for awareness about the topic, then this code was marked ‘1’.  For example: A woman tells the audience that organ donation is a topic which receives to little attention today. She asks people to think about it. | (Yes=1, No=0) | Newly established category following thematic analysis approach [4] |
| Knowledge donations | If the post shares any knowledge related to organ donations or transplants, then this code was marked ‘1’. The category has been slightly adapted from Jiang, X. et al.’s original research and was renamed from ‘organ donation practices’ to ‘knowledge donations’ to more accurately reflect the content themes discussed in our sample.  For example: The post informs the reader that a Xenotransplantation (in this case a heart transplantation from a pig to a human) has been successful in Maryland, USA. | (Yes=1, No=0) | Jiang, X. et. al., 2019 [3] |
| Merchandise/run | If the post shares any information about organ donation merchandise or an organ donor run, then this code was marked ‘1’.  For example: Post informs reader that a doctor participates in the upcoming organ donation run which takes place in Hamburg. | (Yes=1, No=0) | Newly established category following thematic analysis approach [4] |
| Meaning donations | If the post explicitly highlights the meaning of an organ donation for the donor, the recipient or the society, then this code was marked ‘1’.  For example: A wife shares how an organ donation has saved her husband’s life. | (Yes=1, No=0) | Jiang, X. et. al., 2019 [3] |
| Issues/politics | If the post shares either information regarding organ donation politics or on issues with current organ donation systems, then this code was marked ‘1’.  For example: Post introduces the new law which asks physicians to educate their patients about the possibility to register as an organ donor. | (Yes=1, No=0) | Jiang, X. et. al., 2019 [3] |
| Statistical information | If the post shares data or statistical information about organ donations, then this code was marked ‘1’.  For example: The post depicts a chart which illustrates the gap between organ supply and demand in Germany. | (Yes=1, No=0) | Jiang, X. et. al., 2019 [3] |
| Other | If the post shares any information which could not be assigned to any of the category listed above, then this code was marked ‘1’.  For example: The post shares a cartoon of two pigs who are afraid that humans will take away their hearts. | (Yes=1, No=0) | Newly established category following thematic analysis approach [4] |

*Supplementary table 2: Coding of sentiment, messaging strategy, and content themes*

References

1. Boukes M, van de Velde B, Araujo T, Vliegenthart R. What’s the Tone? Easy Doesn’t Do It: Analyzing Performance and Agreement Between Off-the-Shelf Sentiment Analysis Tools. Communication Methods and Measures. 2020;14:83–104. doi:10.1080/19312458.2019.1671966.

2. Cadet, F. T., Aaltonen, P. G., & Kavota, V. (The Advertisement Value of Transformational & Informational Appeal on Company Facebook Pages. Marketing Management Journal. 2017;27:116–30.

3. Jiang X, Jiang W, Cai J, Su Q, Zhou Z, He L, Lai K. Characterizing Media Content and Effects of Organ Donation on a Social Media Platform: Content Analysis. J Med Internet Res. 2019;21:e13058. doi:10.2196/13058.

4. Braun V, Clarke V. Thematic analysis. In: Cooper H, Camic PM, Long DL, Panter AT, Rindskopf D, Sher KJ, editors. APA handbook of research methods in psychology, Vol 2: Research designs: Quantitative, qualitative, neuropsychological, and biological. Washington: American Psychological Association; 2012. p. 57–71. doi:10.1037/13620-004.
